# Cell division in *Corynebacterineae*

**DOI:** 10.3389/fmicb.2014.00132

**Published:** 2014-04-10

**Authors:** Catriona Donovan, Marc Bramkamp

**Affiliations:** Department of Biology I, Ludwig-Maximilians-UniversityMunich, Planegg-Martinsried, Germany

**Keywords:** cell division, cell cycle, Par system, *Corynebacterium glutamicum*, *Mycobacterium tuberculosis*, FtsZ, DivIVA, serine/threonine kinases

## Abstract

Bacterial cells must coordinate a number of events during the cell cycle. Spatio-temporal regulation of bacterial cytokinesis is indispensable for the production of viable, genetically identical offspring. In many rod-shaped bacteria, precise midcell assembly of the division machinery relies on inhibitory systems such as Min and Noc. In rod-shaped *Actinobacteria*, for example *Corynebacterium glutamicum* and *Mycobacterium tuberculosis*, the divisome assembles in the proximity of the midcell region, however more spatial flexibility is observed compared to *Escherichia coli and Bacillus subtilis*. *Actinobacteria* represent a group of bacteria that spatially regulate cytokinesis in the absence of recognizable Min and Noc homologs. The key cell division steps in *E. coli* and *B. subtilis* have been subject to intensive study and are well-understood. In comparison, only a minimal set of positive and negative regulators of cytokinesis are known in *Actinobacteria*. Nonetheless, the timing of cytokinesis and the placement of the division septum is coordinated with growth as well as initiation of chromosome replication and segregation. We summarize here the current knowledge on cytokinesis and division site selection in the *Actinobacteria* suborder *Corynebacterineae*.

## Introduction

Bacterial reproduction by binary fission follows an ordered, programmed sequence of events in which cells increase in mass, replicate genetic material, and segregate newly formed chromosomes before dividing the cytoplasmic and genetic material into two daughter cells. The production of viable progeny necessitates that chromosome replication is initiated at a given cell mass, that the assembly of the cell division machinery is regulated such that it does not precede replication initiation or segregation and, as the divisome matures and as the chromosomes segregate, that membrane invagination is coordinated with cell wall synthesis between the segregated chromosomes. The stages involved in the bacterial cell cycle are not discrete, but rather overlapping and thus often presenting challenges during rapid growth. Chromosome replication initiates at the origin of replication (*oriC*) and is replicated bi-directionally until the replication machineries meet at the terminus region (*terC*). Initiation of chromosome replication is regulated so that the overall rate of DNA synthesis is controlled and is coordinated with cell growth, chromosome segregation as well as cell division (for reviews see Reyes-Lamothe et al., 2012; Costa et al., 2013). Under nutrient rich conditions where cells are growing rapidly, *Bacillus subtilis* and *Escherichia coli* initiate new rounds of replication prior to completion of the previous round, a phenomenon termed multifork replication. However, multifork replication is not ubiquitous. *Caulobacter crescentus*, for example, initiates replication only once per cell cycle. The situation in other bacteria, such as *Corynebacterium glutamicum*, is less clear. Using a marker for the *oriC*, up to four *oriC* foci have been observed in dividing *C. glutamicum* cells, suggesting that multifork replication may not occur (Donovan et al., 2010). However, using flow cytometry up to 16 chromosomes per cell has been recently reported for *C. glutamicum* grown in complex medium (Neumeyer et al., 2013).

A fundamental step in the cycle is the spatial and temporal regulation of cytokinesis, best understood in *B. subtilis* and *E. coli*. These rod shaped bacteria divide with extraordinary accuracy precisely at the midcell. However, this accuracy in division site placement does not hold true for all rod shaped bacteria. Members of the *Actinobacteria* phylum are GC-rich gram positive bacteria, encompassing one of the largest phyla within *Bacteria*. The morphologies of this phylum are diverse, ranging from coccoid (*Rhodococcus*, *Micrococcus*), to rod (*Corynebacterium*, *Mycobacterium*) to even fungal-like hyphae with branched mycelium (*Streptomyces*, *Nocardia*). *Actinobacteria* comprise a number of human pathogens (*Mycobacterium tuberculosis*, *Mycobacterium leprae*, and *Corynebacterium diphtheriae*) as well as non-pathogenic members (*C. glutamicum*, *Streptomyces coelicolor*). The cell wall structure of *Corynebacterineae*, the suborder which is the focus of this review, is highly unusual. In addition to a peptidoglycan layer *Corynebacteria* and *Mycobacteria* contain an outer membrane composed of mycolic acids that is covalently linked to peptidoglycan via the polysaccharide arabinogalactan network (for review see Hett and Rubin, 2008; Favrot and Ronning, 2012). This cell wall structure greatly contributes to resistance to antimicrobial compounds as well as various stress conditions. Aside from the medical importance of *Actinobacteria* as human pathogens, some members such as *C. glutamicum* and *Streptomyces* are industrially central in the production of amino acids and antibiotics, respectively. Regarding biotechnological applications, these organisms have been intensively studied and are now established model organisms (Wieschalka et al., 2013). Nonetheless, relatively little is known about the cell biology of these bacteria.

From initial observations it was clear that *Actinobacteria* differ from the conventional cell biology model organisms such as *B. subtilis* and *E. coli*. Genome sequencing revealed that some *Actinobacteria* lack an actin homolog. *Streptomyces* encode actin homologs that are dispensable for growth (Burger et al., 2000) but are required during exo-spore formation (Mazza et al., 2006). The actin homolog MreB (and its paralogs) plays a critical role in cell elongation in *B. subtilis* and *E. coli*, which in many rod shaped bacteria occurs by intercalation of new peptidoglycan into the cell wall along most of its length (for review see Chastanet and Carballido-Lopez, 2012). Cell division gives rise to hemispherical cell poles in *E. coli*, which are largely inert after formation (De Pedro et al., 2001). In the case of *Actinobacteria*, intercalation of new cell wall material occurs at the cell poles, giving rise to an apical mode of cell elongation (Gray et al., 1990; Daniel and Errington, 2003; Letek et al., 2008b). After maturation of the divisome and completion of cytokinesis, the elongasome (cell wall synthesis machinery) is assembled at the young cell pole and elongation occurs at both poles (Daniel and Errington, 2003). The precise assembly of the elongasome is not completely understood, nor is the existence of two distinct cell division and elongation machineries. In *C. glutamicum* and *M. tuberculosis/smegmatis* as well as other members of the *Actinobacteria* phylum, DivIVA (Wag31 in *Mycobacteriaceae*), has been implicated as an organizer of apical growth (for review see Nguyen et al., 2007; Letek et al., 2008b; Flärdh, 2010).

Compared to established cell division model organisms, striking differences are found in the process of division site selection and cytokinesis in *Actinobacteria*. The incredible precision in division site selection observed for *B. subtilis* and *E. coli* contrasts to *C. glutamicum* and *M. tuberculosis*. It is, therefore, not entirely surprising that the regulatory mechanisms employed by *B. subtilis* and *E. coli* are lacking in *Actinobacteria*. Nonetheless, the genetic integrity of *Actinobacteria* offspring is faithfully maintained.

This review will focus on the current knowledge of cell division in *Corynebacterineae*, with particular emphasis on *C. glutamicum* and *M. tuberculosis*/*M. smegmatis*, and, where appropriate, draw comparisons with other well-established model organisms such as *B. subtilis*, *E. coli* and *C. crescentus*.

## Bacterial cytokinesis

The bacterial cytokinetic machinery, also known as the divisome, is composed of a large number of diverse proteins, many of which are essential for the formation of a functional division apparatus (Table 1) (for review see Adams and Errington, 2009; Lutkenhaus et al., 2012). In *B. subtilis* and *E. coli*, divisome assembly requires the cooperative recruitment of cell division proteins. Assembly of the divisome is initiated by the highly conserved tubulin homolog FtsZ (Bi and Lutkenhaus, 1991). Polymerization of FtsZ into a ring like structure, termed the Z-ring, at the nascent division site forms the cytoskeletal scaffold to which other cell division proteins are recruited. A two stage assembly process for Z-ring formation has been proposed; (i) polymerization of FtsZ into single stranded protofilaments and (ii) lateral interaction between the protofilaments forming stable bundles (Romberg and Levin, 2003). FtsZ rings are dynamic, exhibiting constant subunit exchange with cytosolic molecules (Anderson et al., 2004). Therefore, in order for cell division to occur, the Z-ring must be stabilized at the right time and place during the cell cycle. Both positive and negative regulatory proteins are involved in this process.

**Table 1:** **Putative cell division gene of ***C. glutamicum*** (Cgb), ***M. tuberculosis*** (Mtu) or ***M. smegmatis*** (Msm), ***E. coli*** (Eco), and ***B. subtilis*** (Bsu)**.

**Gene name**	***Cgb***	***Mtu (Msm)***	***Eco***	***Bsu***	**Predicted protein function**	**References**
**EARLY DIVISOME COMPONENTS**						
*ftsZ*	cg0915	rv3101c	b0095	BSU15290	Tubulin homolog, GTPase, polymerizes into Z-ring, recruitment of downstream cell division proteins	Bi and Lutkenhaus, 1991; Dziadek et al., 2003; Ramos et al., 2005; Letek et al., 2007
*ftsA*	?	?	b0094	BSU15280	Actin fold, membrane-associated protein, positive regulator of FtsZ assembly, and membrane association of Z-ring	Beall and Lutkenhaus, 1992; Pichoff and Lutkenhaus, 2005
*zipA*	cg2496/cg3203	rv2345/rv3835	b2412	–	Membrane-associated protein, positive regulator of Z-ring assembly	Hale and De Boer, 1997; Slayden et al., 2006
*zapA*	–	–	b2910	BSU28610	Positive regulator of FtsZ assembly, promotes Z-ring polymerization and protofilament bundling	Gueiros-Filho and Losick, 2002
*zapB*	*–*	–	b3928	–	Interacts with ZapA and FtsZ, stabilize Z-ring via ZapA	Ebersbach et al., 2008; Galli and Gerdes, 2010
*zapC*	–	–	b0946	–	Suppresses FtsZ GTPase activity and promotes lateral association of FtsZ polymers	Durand-Heredia et al., 2011; Hale et al., 2011
*zapD*	–	*–*	b0102	–	Positive regulator of FtsZ assembly, promotes Z-ring polymerization and protofilament bundling	Durand-Heredia et al., 2012
*ftsK*	cg2158	rv2748c (MSMEG2690)	b0890	BSU29800 BSU16800 (*spoIIIE*)	Membrane protein, ATPase, DNA translocator at the division septum, role in chromosome segregation and septum formation, SpoIIIE is a DNA translocator during sporulation in *B. subtilis*	Wu and Errington, 1994; Begg et al., 1995; Singh et al., 2013
*ezrA*	–	–	–	BSU29610	Integral membrane protein, negative regulator of Z-ring assembly, plays a role in midcell localization of Z-rings	Levin et al., 1999; Haeusser et al., 2004
*sepF*	cg2363	rv2147c	–	BSU15390	Putative positive regulator of cell division, promoting Z-ring formation	Hamoen et al., 2006; Ishikawa et al., 2006; Letek et al., 2008a; Al-Khafaji, 2013
*ftsEX*	cg0914	rv3102c	b3463	BSU35260	Putative role in promoting Z-ring assembly, Regulator of peptidoglycan hydrolases. FtsX recruits EnvC to divisome in *E. coli*	Schmidt et al., 2004; Mir et al., 2006; Garti-Levi et al., 2008; Yang et al., 2011
	cg0915	rv3101c	b3462	BSU35250		
**LATE DIVISOME COMPONENTS**						
*ftsB (divIC*)	cg1112^#^	rv1024^*^	b2748	BSU00620	Bitopic membrane protein, forms complex with FtsL and FtsQ in *E. coli* (FtsL and DivIB in *B. subtilis*), links early membrane-associated divisome complex with cytoplasmic components, also likely links divisome with peptidoglycan synthesizing machinery	Levin and Losick, 1994; Buddelmeijer et al., 2002; Al-Khafaji, 2013
*ftsQ (divIB)*	cg2367	rv2151*c*	b0093	BSU15240	Transmembrane protein, forms complex with FtsL and FtsB in *E. coli* (FtsL and DivIC in *B. subtilis*), bridges early membrane-associated divisome complex with cytoplasmic components, also likely links divisome with peptidoglycan synthesizing machinery. In *M. tuberculosis* FtsQ interacts with CrgA	Yi et al., 1985; Beall et al., 1988; Harry and Wake, 1989; Plocinski et al., 2011
*ftsL*	cg2376^**^	Rv2164c^**^	b0083	BSU15150	Transmembrane protein, forms complex with FtsB and FtsQ in *E. coli* (DivIC and DivIB in *B. subtilis*), bridges early membrane-associated divisome complex with cytoplasmic components, also likely links divisome with peptidoglycan synthesizing machinery	Guzman et al., 1992; Daniel et al., 1998; Valbuena et al., 2006
*ftsW*	cg2370	rv2154c	b0089	BSU14850	SEDS member, mediates precursor translocation across the membrane for cognate transpeptidase, bridges cell division and septal peptidoglycan synthesis	Datta et al., 2002, 2006; Mercer and Weiss, 2002; Valbuena et al., 2007; Real et al., 2008; Gamba et al., 2009; Sieger et al., 2013
				BSU15210 (*spoVE*)		
*ftsI*/*pbp3* (*pbp2b*)	cg2375	rv2163c	b0084	BSU15170	Essential HMW-PBP transpeptidase, localized at the septum, interacts with the FtsZ/FtsW complex in *C. glutamicum*, ChiZ and FtsQ in *Mycobacterium*. In *B. subtilis* septal localization depends on FtsL/DivIC/B localization and in *E. coli* FtsW bridges interaction of FtsI with FtsQLB	Weiss et al., 1999; Daniel et al., 2000; Chauhan, 2006; Valbuena et al., 2006, 2007; Slayden and Belisle, 2009
*ftsN*	–	*–*	b3933	–	Bitopic membrane protein, interacts with both early (e.g., FtsA, ZapA) and late cell division proteins (e.g., FtsQLB, FtsW) essential cell division protein, speculated role in coordinating cell division and peptidoglycan synthesis	Ursinus et al., 2004; Gerding et al., 2009; Arends et al., 2010
*crgA*	cg0055	rv0011c	–	–	Localizes to division septum in an FtsZ-dependent manner, interacts with FtsI, FtsQ, PbpA in *Mycobacterium*	Plocinski et al., 2011
*cwsA*	–	rv0008c	–	–	Putative Wag31 topology factor, lack of *cwsA* and *crgA* leads to cell division and cell wall integrity defect in *Mycobacterium*	Plocinski et al., 2012
**REGULATION OF CYTOKINESIS**						
*minC*	*–*	–	b1176	BSU28000	Inhibitor of FtsZ, interacts with MinD	De Boer et al., 1989; Levin et al., 1992
*minD*	–	–	b1175	BSU27990	Membrane-associated ATPase, brings MinC to the membrane	De Boer et al., 1989; Levin et al., 1992
*minE*	–	–	b1174		Topological factor that stimulates pole-to-pole oscillation of MinCD in *E. coli*	De Boer et al., 1989; Raskin and De Boer, 1999
*minJ*	–	–	–	BSU35220	Adaptor protein linking DivIVA and the MinCD complex in *B. subtilis*	Bramkamp et al., 2008; Patrick and Kearns, 2008
*divIVA*	cg2361	rv2145c (*wag31*)	–	BSU15420	Component of the Min system in *B. subtilis*. Component of the apical growth machinery in *C. glutamicum* and *M. tuberculosis*	Cha and Stewart, 1997; Edwards and Errington, 1997; Kang et al., 2008; Letek et al., 2008b
*noc/slmA*	?	?	b3641 (*slmA*)	BSU40990 (*noc*)	Nucleoid occlusion effector proteins, inhibit Z-ring assembly over the nucleoid	Wu and Errington, 2004; Bernhardt and De Boer, 2005
*pldP*	cg1610	rv1708	–	–	Putative spatial regulator of cell division, member of the ParA/MinD protein family	Donovan et al., 2010
*mciZ*	*–*	–	–	BL02920*^§^*	Small peptide, inhibits Z-ring assembly during sporulation in *B. subtilis*	Handler et al., 2008
*clpX*	cg2620	rv2457c	b0438	BSU28220	Putative negative regulator of cell division, inhibits Z-ring formation under stress conditions	Weart et al., 2005; Camberg et al., 2009; Haeusser et al., 2009; Dziedzic et al., 2010; Sugimoto et al., 2010
*divS^§§^*	cgR_1759^***^ (cg2113, *C. glutamicum* ATCC 13032)	rv2719c (*chiZ*)	b0958 (*sulA*)	BSU17860 (*yneA*)	Induced upon DNA damage, inhibits Z-ring formation, ChiZ is a cell wall hydrolase and interacts with FtsI and FtsQ	Huisman et al., 1984; Bi and Lutkenhaus, 1993; Kawai et al., 2003; Chauhan et al., 2006; Ogino et al., 2008
*ugtP*	?	?	?	BSU21920	Metabolic sensor that inhibits Z-ring assembly in response to high UDP-glucose availability (nutrient rich conditions)	Weart et al., 2007
**CELL SEPARATION**						
*amiC*	cg3424 (*cwlM*)	rv3915 (*cwlM*)	b2817	BSU09420 (*lytE*)	N-acetylmuramoyl-l-alanine amidase, septal peptidoglycan hydrolysis to separate daughter cells, cleaves stem peptides and N-acetylmuramic acid components of glycan stands, septal recruitment dependent on FtsN	Heidrich et al., 2001; Bernhardt and De Boer, 2003; Deng et al., 2005; Priyadarshini et al., 2007
				BSU09370 (*lytF*)		
*envC*	cgR_1596^***^ (cg1735, *C. glutamicum* ATCC 13032)	rv1477 (*ripA*)	b3613	BSU34800 (*cwlO*)	LytM domain containing, activator of amidases AmiC (and AmiB) (*E. coli*), regulated by FtsEX. RipA interacts with the lytic transglycosylase Rpf, depletion of *ripA* leads to chains of cells	Bernhardt and De Boer, 2004; Hett et al., 2008; Tsuge et al., 2008; Yang et al., 2011; Meisner et al., 2013

* M. tuberculosis H37Rv,

# shows weak homology, ? not known,

§ B. licheniformis,

§§ Not homologs but all have analogous function as septation inhibitors,

** Weak homology but similar topology,

*** *C. glutamicum R*.

### Divisome components and assembly

In *B. subtilis* and *E. coli*, FtsZ levels do not fluctuate during the cell cycle, thus Z-ring assembly must be subject to regulation (Weart and Levin, 2003), contrary to the situation in *C. crescentus* (Hottes et al., 2005). The cellular signals required for Z-ring assembly are not completely understood, however a slight increase in FtsZ level results in multiple, non-productive Z-rings, while *ftsZ* overexpression leads to filamentous cells, suggesting that the assembly of FtsZ ring formation is controlled by both positive and negative factors. The impact of altering FtsZ levels is more severe in *C. glutamicum* and *M. tuberculosis*, suggesting that cell division in these organisms is sensitive to the intracellular levels of FtsZ (Dziadek et al., 2003; Ramos et al., 2005; Letek et al., 2007). Overexpression of *ftsZ* in *C. glutamicum* gives rise to altered morphology as well as altered nucleoid localization (Letek et al., 2007).

Assembly of the divisome is a two-step process with a time delay of approximately 20% of the cell cycle between the two steps. After recruitment of FtsZ to the nascent division site, a group of early recruited proteins facilitate anchoring of FtsZ to the membrane and stabilization of the Z-ring (in *E. coli*—FtsA, ZipA, ZapA, FtsK and in *B. subtilis*—FtsA, ZapA, EzrA, SepF, ClpX) (for review see Huang et al., 2013). The late recruited divisome components are mostly membrane anchored proteins involved in the maturation of the divisome and recruitment appears to follow a hierarchical interdependent pattern (in *E. coli*—FtsB, FtsQ, FtsL, FtsW, FtsI, FtsN and in *B. subtilis*—DivIB, DivIC, FtsL, Pbp2b, FtsW, SpoIIIE) (for review see Margolin, 2005; Lutkenhaus et al., 2012). In the final steps, septal peptidoglycan synthesis and constriction of the cell envelope are triggered, before murein hydrolases separate the daughter cells.

### Membrane tethering and stabilization of the Z-ring

Given that FtsZ itself does not have affinity for the membrane, additional factors to stably tether the Z-ring to the cell membrane are required. In *E. coli*, the actin homolog FtsA targets FtsZ to the membrane via an amphipathic helix (Pichoff and Lutkenhaus, 2005). In *B. subtilis ftsA* mutants are extremely filamentous (Beall and Lutkenhaus, 1992). In the absence of FtsA, ZipA is essential to tether Z-rings to the *E. coli* membrane (Hale and De Boer, 1997). Recent evidence suggests that FtsA is the dominant Z-ring tethering factor and ZipA may function in the disruption of FtsA self-interaction allowing FtsA to interact with and tether FtsZ rings to the membrane (Pichoff et al., 2012). Similar to other early recruited divisome components, FtsA is required for the recruitment of downstream cell division proteins. In *E. coli*, two cytoplasmic components of the division machinery, ZipA and ZapA, have been reported to promote lateral bundling of the GTP bound FtsZ protofilaments, stabilizing the Z-rings (Hale and De Boer, 1997; Liu et al., 1999; Raychaudhuri, 1999; Gueiros-Filho and Losick, 2002). *In vitro* evidence has recently been presented proposing that ZipA restructures FtsZ polymers leading to the formation of higher ordered structures (Mateos-Gil et al., 2012). Although not essential under normal lab growth conditions, *B. subtilis* cells lacking *zapA* are found to be synthetic lethal in the absence of *ezrA* (see below) or in cells with reduced FtsZ levels (Gueiros-Filho and Losick, 2002).

Genome wide blasts to identify conventional FtsZ interacting proteins revealed that *C. glutamicum*, as well as other members of the *Actinobacteria* phylum, lack the eminent positive regulators (FtsA, ZipA, or ZapA) found in the conventional rod-shaped bacteria (Letek et al., 2007; Hett and Rubin, 2008). However, transcription profiling of *M. tuberculosis* cells inhibited for septum formation revealed two potential orthologs of ZipA (Rv2345 and Rv3835) (Slayden et al., 2006). Homologs of the putative *M. tuberculosis* cell division proteins can also be found in *C. glutamicum* (Cg2496 and Cg3203, respectively), however an involvement in cell division remains to be validated in both organisms.

Another FtsZ interacting partner is SepF, a positive regulator of cell division in *B. subtilis* (Hamoen et al., 2006). SepF associates with the membrane through an N-terminal amphipathic helix and recruits FtsZ by binding to the C-terminal domain of FtsZ (Król et al., 2012; Duman et al., 2013). Mutants of *sepF* are viable, however cells have a mild division defect (Hamoen et al., 2006; Król et al., 2012). SepF is widely conserved in gram positive bacteria, with homologs in both *C. glutamicum* (Cg2363) and *M. tuberculosis* (Rv2147c) (Letek et al., 2007). A more detailed study is required before these homologs can be assigned a specific function or role in the *C. glutamicum* divisome complex.

The FtsEX complex, which structurally resembles an ABC transporter, is recruited relatively early to the divisome (Schmidt et al., 2004). FtsE encompasses the ATPase domain while FtsX forms the transmembrane domain of the complex (Arends et al., 2009). In *E. coli*, FtsEX is essential only under low osmolarity conditions, where it functions to regulate peptidoglycan hydrolases (Schmidt et al., 2004). Midcell localization of FtsX is achieved through interactions with FtsA and FtsQ, while FtsE interacts directly with FtsZ in *E. coli* (Schmidt et al., 2004; Corbin et al., 2007). In *B. subtilis*, FtsEX is found all around the cell membrane and plays a role in the spatio-temporal regulation of sporulation, although the precise mechanism is not known, yet (Garti-Levi et al., 2008). It has recently been proposed that FtsEX of *Streptococcus pneumoniae* and *B. subtilis* functions in signal transduction coupling peptidoglycan hydrolysis and cell division (Meisner et al., 2013; Sham et al., 2013). More recently, FtsEX was shown to regulate the activity of CwlO (a cell wall hydrolytic enzyme) (Dominguez-Cuevas et al., 2013; Meisner et al., 2013). FtsEX of *C. crescentus* is recruited to the nascent division site prior to FtsA, and hence is speculated to play a role in tethering of Z-rings to the membrane (Goley et al., 2011). The genomes of *C. glutamicum* and *M. tuberculosis* contain FtsEX homologs, however relatively little is known about their function in the respective organisms, not to mention a potential role in cell division. In *M. tuberculosis*, FtsEX exists as a complex found in the membrane (Mir et al., 2006), however the precise subcellular localization is not known, yet. It is tempting to speculate that in bacteria lacking FtsA, FtsEX plays a role in promoting FtsZ assembly and stability at the impending division site.

### Negative regulation of divisome assembly

In pre-divisional *B. subtilis* cells, EzrA is found throughout the membrane and has been proposed to prevent FtsZ assembly at inappropriate sites other than mid-cell, impacting on spatial regulation of the Z-rings. At the onset of Z-ring assembly EzrA becomes concentrated at the nascent division site where it is speculated to interact directly with FtsZ playing a role in remodeling of the division rings (Levin et al., 1999; Haeusser et al., 2004). In cells lacking *ezrA*, extra Z-rings can form because the critical EzrA concentration required for FtsZ assembly is reduced. Thus, EzrA ensures that Z-rings form only once per cycle, or it might prevent Z-ring assembly at the cell poles (Levin et al., 1999). In *Staphylococcus aureus*, EzrA appears to be important for maintaining correct cell size (Jorge et al., 2011). Additionally, these cells lacking *ezrA* have delocalized FtsZ and PBP's, along with septa aberrations (Jorge et al., 2011). EzrA homologs have not been identified in *C. glutamicum* or *M. tuberculosis*, however as outlined below there are a number of proteins that might function analogous to EzrA.

The highly conserved ClpX protein has been shown to play a role in modulating cell division in a number of bacteria. In *B. subtilis*, the ClpX chaperone (the substrate recognition domain of the ClpXP protease) inhibits Z-ring formation in an ATP hydrolysis independent manner (Weart et al., 2005). It was proposed that ClpX sterically interferes with FtsZ assembly (Weart et al., 2005; Haeusser et al., 2009). The ClpXP protease has been implicated in regulating cell division in a number of organisms. In *C. crescentus*, this complex degrades the master regulator, CtrA, and thereby modulates the expression of cell division genes, including *ftsZ*, in a cell cycle dependent manner. In *E. coli*, ClpXP has been proposed to modulate FtsZ polymer dynamics, either through degradation of the FtsZ monomers and polymers (Camberg et al., 2009) or by disassembling FtsZ polymers (Sugimoto et al., 2010). In *M. tuberculosis*, ClpX (Rv2457c) has been reported to interact with FtsZ independent of the oligomeric state of FtsZ or its association with other proteins (Dziedzic et al., 2010). *In vivo*, ClpX and FtsZ colocalize, however this colocalization was more prominent with polar FtsZ foci (Dziedzic et al., 2010). ClpX is upregulated under stress conditions, such as exposure to antibiotics or intracellular growth of *M. tuberculosis* and has been proposed to bind FtsZ monomers inhibiting assembly of a functional Z-ring, delaying cell division under stress conditions. Alternatively, polar localized ClpX could function to prevent reassembly of a polar Z-ring (Dziedzic et al., 2010). In *M. tuberculosis*, it is not clear if ClpX is essential under normal growth conditions. Reduced levels of the essential ClpX protein appears to alter FtsZ ring formation, however this could be an indirect effect (Dziedzic et al., 2010). A homolog of ClpX is present in the *C. glutamicum* genome (cg2620) (Table 1).

DNA damage leads to inhibition of Z-ring formation allowing cells to repair damaged DNA without guillotining the chromosome or produce offspring with damaged genomes. The SOS response induced under such conditions is controlled by RecA and LexA proteins. Upon DNA damage, RecA is activated and inhibits the DNA-binding capacity of LexA by proteolytic cleavage LexA is a suppressor of SOS response genes under normal growth conditions (Little et al., 1980; Horii et al., 1981). In *C. glutamicum*, the expression of up to 48 genes is controlled by LexA, many of unknown function (Jochmann et al., 2009). In *C. glutamicum* R, an unrelated protein DivS (CgR_1759, *C. glutamicum* ATCC13032 Cg2113) is induced in response to DNA damage (Ogino et al., 2008). Induction of DNA damage and expression of *divS* by mitomycin C treatment or overexpression of *divS* suppresses cell division by reduced Z-ring formation (Ogino et al., 2008). The morphological defects of a *divS* overexpression mutant, elongated cells with growth from ectopic sites (Ogino et al., 2008), are similar to mutants with reduced FtsZ levels (Ramos et al., 2005). These morphological defects could not be complemented by overexpression of *ftsZ*, suggesting that suppression of cell division does not involve a direct interaction with FtsZ.

In *Mycobacterium*, the cell wall hydrolase ChiZ (Rv2719c) is induced upon DNA damage and acts as a negative regulator of cell division (Chauhan, 2006). When overexpressed fewer FtsZ rings per cell were observed. In the absence of *chiZ*, FtsZ rings were stabilized. An interaction of ChiZ with FtsI and FtsQ was observed, but not with FtsZ or Wag31 (DivIVA in *C. glutamicum*). ChiZ does not influence the localization of FtsI, FtsQ, or Wag31, suggesting that recruitment and function of ChiZ is downstream of FtsI/Q assembly to the divisome. ChiZ has an extracellular LysM domain, however this does not appear to be necessary for its function (Chauhan, 2006).

In *E. coli*, the SulA is induced in the SOS response (Huisman et al., 1984; Bi and Lutkenhaus, 1993) and inhibits cell division by binding directly to FtsZ and inhibiting its polymerization (Mukherjee et al., 1998). In *B. subtilis*, YneA, a transmembrane protein with an extracellular LysM peptidoglycan binding domain, is induced upon DNA damage (Kawai and Ogasawara, 2006). YneA does not interact directly with FtsZ and during the SOS response Z-ring formation is reduced as opposed to complete abolishment. The *C. glutamicum* DivS does not share significant homology to YneA; however the genomic organization is similar, with the *lexA* gene found in close proximity to SOS-induced genes.

### Coordination of cell division with septal peptidoglycan synthesis

The recruitment and stabilization of the Z-ring at the nascent division site must be followed by divisome constriction, meaning septal peptidoglycan synthesis must be initiated so that invagination of the cell envelope leads to synthesis of new cell poles and separation of the daughter cells. The precursor molecules required for peptidoglycan synthesis must be translocated across the membrane by the action of lipid II flipases before incorporation into the inner membrane by the action of penicillin-binding proteins (Pbp). The relatively late recruited divisome protein FtsW is a member of the SEDS (shape, elongation, division, and sporulation) protein family that mediates precursor translocation across the membrane (Ehlert and Höltje, 1996; Mohammadi et al., 2011). FtsW is normally found associated with its cognate Pbp. The *E. coli* FtsW localizes Pbp3 (FtsI) to the divisome, while divisome recruitment of FtsW is dependent on FtsL and FtsQ (Mercer and Weiss, 2002). In *M. tuberculosis*, FtsW is a central component in a complex that bridges cell division and septal peptidoglycan synthesis (Datta et al., 2002, 2006). Four aspartic acids at the carboxyl terminus of FtsZ are required for FtsW interaction, while the C-terminal region of Pbp3 (FtsI) appears to be necessary for interaction with FtsW (Datta et al., 2002, 2006). Given that an FtsZ variant in which the three aspartic acids of the C-terminus of FtsZ were mutated to alanine could not be expressed as the only copy, it has been proposed that this region of FtsZ is essential for FtsZ activity and cell division in *M. tuberculosis* (Rajagopalan et al., 2005). When expressed in trans, the FtsZ mutant variant associated with the Z-ring indicating that self-interaction was not impaired. Conditional inactivation of *M. smegmatis* FtsW did not impede targeting of FtsZ to the nascent division site, however Pbp3 (FtsI) fails to localize (Datta et al., 2006). Septation was compromised in cells with reduced FtsW levels in addition to longer cells, with multiple, probably non-functional, Z-rings positioned between segregated chromosomes.

In *C. glutamicum*, FtsW localizes to the division septa (Sieger et al., 2013) where it interacts with FtsZ directly (Valbuena et al., 2007). However, the temporal order of FtsW recruitment to the divisome is not yet known. Similar to FtsW, FtsI is essential in *C. glutamicum* and localizes to the division septum likely in an FtsW-dependent manner, similar to *E. coli* and *B. subtilis* (Weiss et al., 1999; Daniel et al., 2000; Valbuena et al., 2006, 2007). Reduced expression of *ftsI* causes severe morphological defects and also induces the expression *of* several genes, including *divIVA*, which is involved in apical growth (Valbuena et al., 2006).

### Cell division proteins specific to *Actinobacteria*

Originally identified and characterized in *S. coelicolor*, CrgA is a protein that has been implicated in cell division in *Actinobacteria*. In *M. tuberculosis*, altered expression of *crgA* led to cell morphological as well as division defects, but did not affect FtsZ levels (Plocinski et al., 2011). Localization studies of the *M. tuberculosis* CrgA in *M. smegmatis* revealed that CrgA localizes to the division septum in an FtsZ-dependent manner but does not influence Z-ring localization (Plocinski et al., 2011). Interaction with FtsZ could be mapped to the N-terminal domain of CrgA. Further interaction analysis revealed that CrgA interacts with FtsI, FtsQ, and PbpA, and is required for FtsI septal localization. CrgA might be required for the recruitment of components of the peptidoglycan synthesis machinery, such as FtsI and PbpA (transpeptidases involved in peptidoglycan cross-linking during septum synthesis), to the mature division site as synthesis of the invagination septum begins to occur. *C. glutamicum* also encodes a CrgA homolog (Cg0055), however this protein has not been investigated to date.

Further evidence that CrgA synchronizes cell division and cell septation came from interactions with a novel mycobacterial protein, CwsA. This small transmembrane protein localizes to the cell poles and division septum. Single deletion of *cwsA* did not influence Z-ring localization, however combined deletion of *cwsA* and *crgA* did compromise Z-ring assembly and cell wall integrity (Plocinski et al., 2012). In addition, deletion of *cwsA* negatively influenced cell growth and morphology, but more interestingly has been reported to alter the localization of Wag31 (DivIVA in *C. glutamicum*) (Plocinski et al., 2012), the orchestrator of apical growth in *Actinobacteria*. So far, the only evidence that CwsA and Wag31 interact came from a bacterial two hybrid analysis (Plocinski et al., 2012).

Positive regulators of FtsZ polymerization have also been described for *Streptomyces coelicolor*. During vegetative growth FtsZ is dispensable but spore development defects are observed (McCormick et al., 1994; McCormick and Losick, 1996). Two novel positive regulators of FtsZ, SsgA, and SsgB, were identified recently in *S. coelicolor* (Willemse et al., 2011). SsgA and SsgB localize independently of FtsZ (Willemse et al., 2011), however in their absence sporulation is blocked (Van Wezel et al., 2000; Keijser et al., 2003). In young aerial hyphae, SsgA localizes to alternating sides of the aerial hyphae membrane, recruiting SsgB to these sites. SsgB is speculated to tether the FtsZ spiraling filaments to the membrane of sporulating cells.

## Division site selection

Essentially, FtsZ lays the foundation for the subcellular localization and symmetry of the division site. Aside from protein-protein interactions that stabilize Z-ring formation and assembly of the cytokinetic machinery, FtsZ is also subject to spatial regulation.

### Spatial regulation of septum placement

Two systems that employ negative regulation to spatially control division site selection have been identified and extensively studied in *E. coli* and *B. subtilis*. The nucleoid occlusion (NO) system acts as an anti-guillotine system to protect the nucleoid from aberrant Z-ring formation. The second regulatory system employed by *E. coli* and *B. subtilis* is the Min system. The Min system of *E. coli* consists of MinCDE, which collectively prevents division form occurring at the cell poles (for review see Lutkenhaus, 2007). This pole-to-pole oscillating system involves MinE activation of MinD ATPase, resulting in MinD release from the membrane which is closely followed by MinC, the actual inhibitor of FtsZ polymerization, The Min system of *B. subtilis* is less dynamic than that of *E. coli* (for review see Bramkamp and Van Baarle, 2009). Here, the Min system consists of the topological determinant DivIVA, an adaptor protein MinJ, MinD, and the FtsZ inhibitor MinC. In the absence of MinCD or MinJ disassembly of the divisome is impaired, resulting in the initiation of a new round of cytokinesis close to the original site of division, leading to rows of minicells (Van Baarle and Bramkamp, 2010). Thus, the Min system in *B. subtilis* is also required to disassemble the cytokinetic machinery.

### Spatial and temporal regulation of division and chromosome segregation by the MinD/ParA protein family

*Corynebacterineae* do not encode a Min system, however MinD related proteins are found in *C. glutamicum* and *M. tuberculosis*, for example ParA proteins. The highly conserved ParAB system has been shown in a number of organisms to be necessary for segregation of the *oriC* regions, as well as overall chromosome organization with respect to the *oriC*/*terC* localization (Wang et al., 2013). The tripartite partitioning system comprises of two *trans*-acting proteins, encoded in an operon (*parA* and *parB*, respectively), and *cis*-acting “centromere-like” elements. After initiation of chromosome replication, the ParB protein binds the centromere-like element *parS* forming a nucleoprotein complex (Funnell, 1988). Interaction with ParB proteins stimulates the ATPase activity of the ParA Walker type P-loop ATPase activating the molecular switch that regulates ParA localization and activity. In the ATP bound form ParA dimerizes and binds non-specifically to DNA while ParA-ADP does not bind DNA (Leonard et al., 2005; Murray and Errington, 2008). In *B. subtilis*, ParA-ADP (Soj) regulates DNA replication initiation by direct interaction with DnaA (Scholefield et al., 2012). Although still under debate, ParA has been implicated in providing the driving force, either through a pulling or pushing mechanisms, for *oriC* segregation (Ptacin and Shapiro, 2010; Schofield et al., 2010; Vecchiarelli et al., 2010).

In *C. crescentus*, where cell division is strictly coordinated with cell division, the Par system is essential (Mohl and Gober, 1997). On the contrary, *par* deletion mutants in *B. subtilis* exhibit only mild segregation defects (Ireton et al., 1994; Lin and Grossman, 1998; Webb et al., 1998). Mutation of components of the Par system in *C. glutamicum* or *M. tuberculosis* lead to a plethora of phenotypes including variable cell lengths, growth defects, multinucleoid cells as well as anucleate cells (Maloney et al., 2009; Donovan et al., 2010; Ginda et al., 2013).

In *M. smegmatis*, ParA is diffused in the cytoplasm, but additionally localizes as foci that are either unipolar, bipolar, or bipolar with a ParA focus around the midcell region (Ginda et al., 2013). The localization of ParB is somewhat unclear. Ginda and co-workers stated that ParB foci are found positioned in the vicinity of the cell poles, while Maloney and co-workers report ParB foci positioned at the cell poles (Maloney et al., 2009). Similarly, *C. glutamicum* ParA localizes as foci to the cell poles and forms patches over the chromosome, while ParB forms polar localized foci (Donovan et al., 2010). In *C. glutamicum* and *M. smegmatis*, cells lacking *parA* result in the production of DNA-free cells, extra ParB foci are produced and distribution of these ParB foci is altered, in addition to altered septum placement (Donovan et al., 2010; Ginda et al., 2013). These phenotypes would suggest that the Par system additionally plays a role in regulating replication initiation and division site positioning. Even though the division site placement is more variable in *C. glutamicum* and *M. smegmatis/M. tuberculosis* compared to *B. subtilis* and *E. coli*, regulatory mechanisms must exist as the divisome is positioned within a range of the precise midcell in the vast majority of cells (Aldridge et al., 2012; Joyce et al., 2012; Donovan et al., 2013; Santi et al., 2013). It has been reported that the divisome often assembles over the (unsegregated) nucleoid suggesting that a NO system does not exist in *C. glutamicum* or *M. tuberculosis* and also that DNA translocases play an important role in the removal of DNA from the invaginating division septum (Ramos et al., 2005; Donovan et al., 2013; Singh et al., 2013).

The generation of unequal sized daughter cells after cell division in *M. smegmatis* has been proposed to be the consequence of asymmetric polar growth after precise positioning of the nascent division septum at midcell (Aldridge et al., 2012; Joyce et al., 2012). On the contrary, two recent reports suggested that daughter cell asymmetry results from acentric placement of the Z-ring slightly closer to the new cell pole and is not influenced by asymmetric polar growth (Santi et al., 2013; Singh et al., 2013). In *C. glutamicum*, asymmetric polar cell growth has been observed with slightly elevated growth at the old pole, however the localization of FtsZ on a single cell level has not been investigated, to date (Sieger et al., 2013).

Single cell analysis of live *C. glutamicum* cells revealed that cell division does not normally occur close to the cell poles (Donovan et al., 2013). Given that the *oriC* region of the chromosome is speculated to be tethered at the cell pole through an interaction between the ParB-*oriC* nucleoprotein complex and the polar growth determinant DivIVA, divisions close to the cell poles would ultimately lead to chromosome guillotining (Donovan et al., 2012; Ginda et al., 2013). It should be noted that *M. smegmatis* polar ParA foci interacts with Wag31, however the consequence of this interaction is not clear, yet (Ginda et al., 2013). When the organization of the *C. glutamicum* nucleoid is compromised by mutation in the Par system, the placement of the division site is much more varied (Donovan et al., 2013). In addition, the growth rate is impaired in cells lacking *parA* or *parB* and also the timing of division events is much more variable, suggesting that chromosome segregation is coupled to cell growth and division (Donovan et al., 2013). This is not the case in *B. subtilis par* mutants, likely as a consequence of overlapping systems that ensure the fidelity of spatial regulation of the division septum.

## ParA-like proteins and cell division

Not all bacteria that divide in a medial fashion encode a Min or NO system, for example *C. crescentus*. In *C. crescentus*, MipZ, which is a member of the ParA/MinD family, spatially regulates Z-ring assembly by stimulating the GTPase activity of FtsZ and inhibiting polymerization (for review see Thanbichler, 2010). MipZ localizes as a gradient that moves with the replicated sister origin to the old cell pole, where FtsZ is localized. Similar to other members of the ParA/MinD family, regulation of MipZ is nucleotide dependent (Kiekebusch et al., 2012). More recently, another ParA-like protein was implicated in positively regulating division site selection in *Myxococcus xanthus* a rod-shaped δ-proteobacterium. This protein, PomZ localizes the nascent division site before FtsZ and is necessary for recruitment of the FtsZ (Treuner-Lange et al., 2013).

While both *C. glutamicum* and *M. tuberculosis* encode the canonical ParAB partitioning system, *C. glutamicum* encodes one additional ParA-like protein, whereas *M. tuberculosis* has two ParA-like proteins (Maloney et al., 2009; Donovan et al., 2010). The transcription profiling that identified two *M. tuberculosis* ZipA-like proteins additionally identified two MinD/ParA-like proteins (Rv1708 and Rv3660c) (Slayden et al., 2006). One of these proteins (Rv1708) is homologous to the orphan ParA-like protein found in *C. glutamicum* (PldP, Cg1610). The chromosomal location as well as a complete lack of a partner ParB protein would suggest that the orphan ParA-like protein is not, or not strongly, involved in chromosome segregation. A mutant deleted for *pldP* is not significantly impaired in chromosome segregation, however cell lengths are more variable, ranging from significantly longer to small, DNA free minicells (Donovan et al., 2010). The *pldP* mini cell phenotype is reminiscent of *B. subtilis* or *E. coli min* mutants. Lending to the notion that PldP plays a role in cell divisions, expression of *pldP*-*cfp* as a single copy from the native locus shows PldP foci at the nascent division site along with weaker signals that might be a result of binding to the nucleoid. Nonetheless, further work needs to be carried out to establish if PldP plays a role in regulating the localization of the Z-ring.

**Figure 1:**
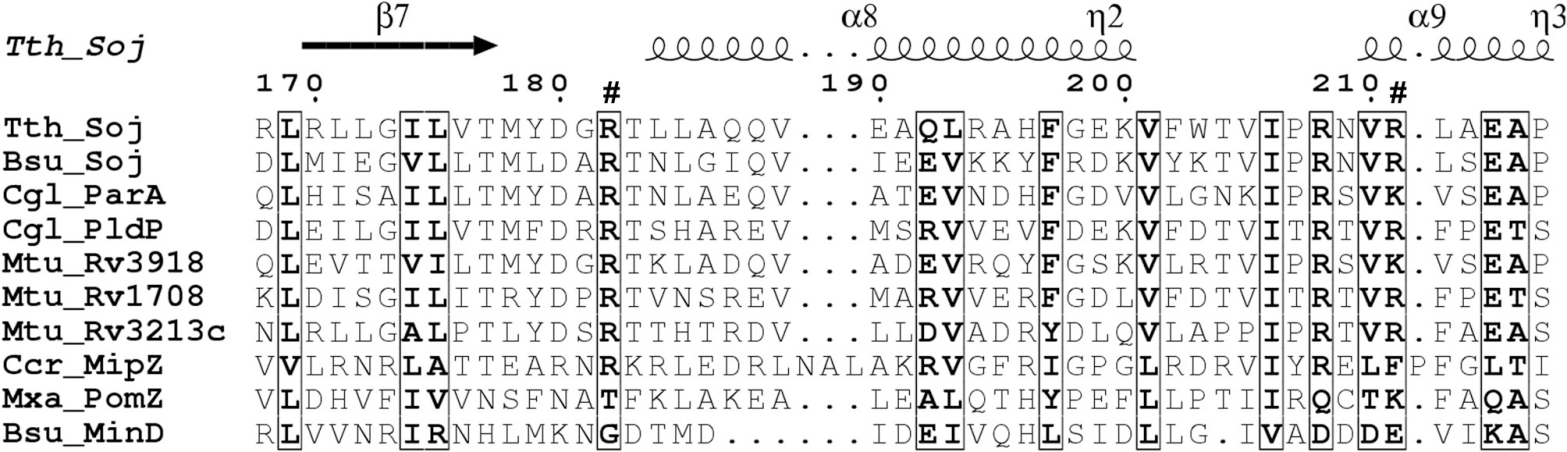
**Alignment of ParA proteins highlighting basic residues involved in DNA-binding**. Surface exposed arginine residues have been shown to be involved in DNA-binding in *B. subtilis Soj* (Hester and Lutkenhaus, 2007). We have aligned sequence of different ParA/MinD proteins using ClustalW2. The alignment was rendered in ESPript (Gouet et al., 2003). Note that *C. glutamicum* ParA and PldP have the conserved positive charges (arrows) identified for DNA-binding in *B. subtilis*, while MinD lacks positive charges at these sites. Bsu, *B. subtilis*; Ccr, *C. crescentus*; Cgl, *C. glutamicum;* Mtu, *M. tuberculosis*; Mxa, *M. xanthus*; Tth, *T. thermophilus*.

Comparison of the PldP sequence with ParA's and MinD's from other organisms reveals that the residues required for ATP-binding and hydrolysis, as well as DNA binding are conserved in PldP (Figure 1). Alternatively, recruitment might be a result of direct protein-protein interactions between PldP and other, as of yet unidentified, divisome components. Overall, members of the ParA/MinD protein family seem to play an important role in regulating cell cycle events, be it chromosome segregation, cell division, or both.

## Regulation of cell division through protein phosphorylation

Reversible protein phosphorylation is a fundamental mechanism to regulate protein activity and is commonly used by bacteria to sense and rapidly respond to changing environmental conditions as well as cell cycle events and control cellular function (Greenstein et al., 2005; Burnside and Rajagopal, 2012). Prokaryotic counterparts of the eukaryotic serine/threonine protein kinases (STPK) have emerged as important regulators of numerous cellular processes, such as cytokinesis as well as regulation of cell growth and shape (Molle and Kremer, 2010). *Actinobacteria* encode varying numbers of kinases, 4 in *C. glutamicum* and 11 in *M. tuberculosis* (Cole et al., 1998; Ikeda and Nakagawa, 2003; Kalinowski et al., 2003). In both *C. glutamicum* and *M. tuberculosis*, two of the kinases (PknA and PknB) are found in an operon with RodA (lipid II flipase) and PbpA (for review see Molle and Kremer, 2010).

The four eukaryotic-like Ser/Thr protein kinases (STPKs) encoded in *C. glutamicum* are designated PknA; PknB, PknL, and PknG, in analog to the Mycobacteria STPK's. With the exception of PknG, all the STPKs are predicted membrane integral proteins (Fiuza et al., 2008; Schultz et al., 2009). Furthermore, PknB and PknL have several PASTA (penicillin-binding protein and serine/threonine kinase associated) domains at their extra-cytoplasmic terminus, probably sensing peptidoglycan components, inducing dimerization and subsequent phosphorylation. In addition, a single gene (*ppp*) coding for a phospho-serine/threonine protein phosphatase was annotated on the *C. glutamicum* genome (Ikeda and Nakagawa, 2003; Kalinowski et al., 2003). In *Mycobacterium* and *Corynebacterium*, the operon encoding *pknA* and *pknB* genes also encodes for proteins involved in cell peptidoglycan synthesis suggesting a functional relationship between the STPK's and cell morphology and division.

The extracellular PASTA domain of *B. subtilis* Ser/Thr kinase PrkC binds peptidoglycan and stimulates germination of spores (Shah et al., 2008). In *M. tuberculosis*, the midcell and polar localization of PknB requires the extracytoplasmic PASTA domain (Mir et al., 2011). This domain has been proposed to bind peptidoglycan precursors or hydrolysis products, leading to a concentration of PknB at regions of the cell where cell growth occurs, namely the cell poles and division septa (Mir et al., 2011). Increased local concentration of PknB might facilitate dimerization and activation, subsequently playing a role in regulating cell growth and division. Both PbpA, a peptidoglycan synthesizing enzyme localized to the septum and important for cell division (Dasgupta et al., 2006), and Wag31 have been identified as substrates of PknB (Kang et al., 2005; Dasgupta et al., 2006). Alterations in the expression level of *pknA* or *pknB* lead to altered morphology, including growth from ectopic sites (Kang et al., 2005).

**Figure 2:**
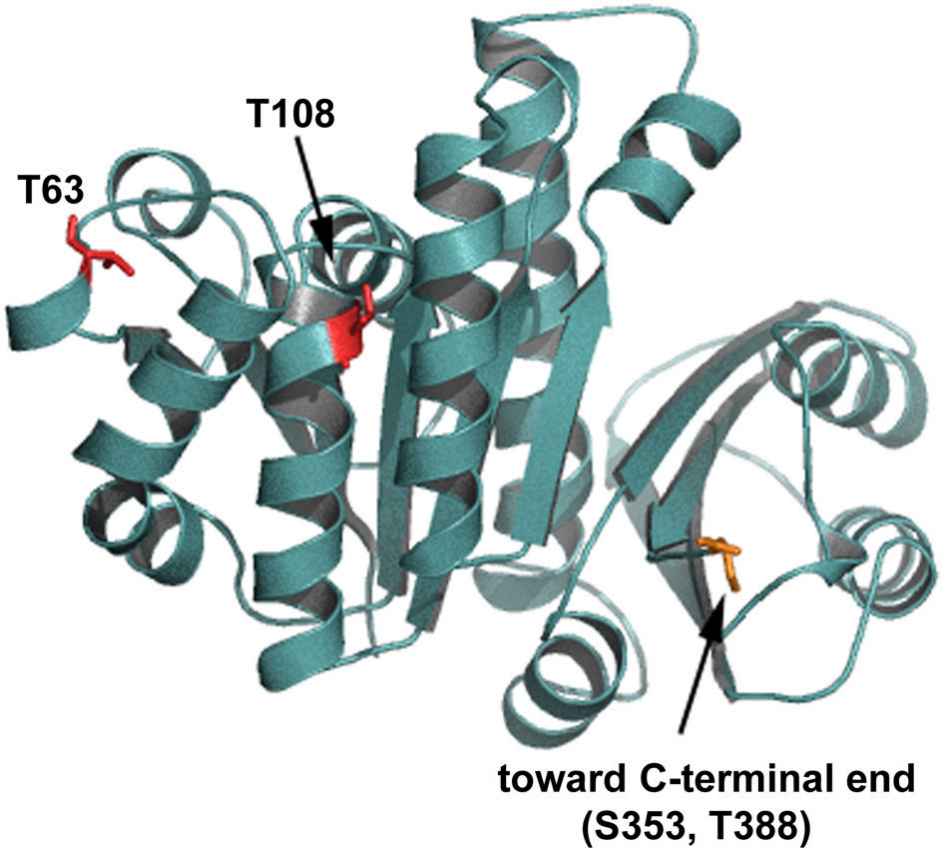
**Putative PknA phosphorylation sites on FtsZ**. Shown is a homology model of *C. glutamicum* FtsZ. The residues phosphorylated by Ser/Thr protein kinases are shown in red and indicated. Residue T108 is involved in nucleotide binding/hydrolysis, T63 is at the interface of the FtsZ oligomerization domain. Residues S353 and T388 are located toward the C-terminal end. The C-terminal end is not depicted in this model.

Initial characterization of *C. glutamicum* STPKs revealed that deletion mutants, in all combinations, are viable with the exception of a *pknAB* mutant (Schultz et al., 2009). While growth rates of the single STPK's mutants are comparable to wild type, the mutant cells are elongated suggesting a role in cytokinesis and cell morphogenesis. This was particularly evident in cells lacking *ppp*, which have severe morphology and growth defects (Schultz et al., 2009). In line with these observations, apical growth is abolished upon overexpression of *pknA* or *pknB* (Fiuza et al., 2008). The cell division protein FtsZ was identified as an *in vivo* target for the phosphatase Ppp and *in vitro* phosphorylation by PknA, PknB, and PknL (Schultz et al., 2009). Four phosphorylation sites were identified (FtsZ^T108^ phosphorylated by PknA and FtsZ^T63^, FtsZ^S353^, and FtsZ^T388^ phosphorylated by PknL). If the sites of FtsZ phosphorylation are mapped onto a 3D model of *C*. *glutamicum* FtsZ it becomes obvious that all four major phosphorylation sites could affect different functions of FtsZ (Figure 2). Residue T108 is involved in nucleotide binding/hydrolysis, T63 is at the interface of the FtsZ oligomerization domain and residues S353 and T388 are located toward the C-terminal end. It is easy to envision that the different sites may contribute to specific cellular functions. The C-terminal part of FtsZ is most likely an interaction domain to recruit downstream proteins to the site of septation (Haney et al., 2001). Although not completely understood, it is clear that STPK play an important regulatory role in cell division in *Actinobacteria* (for review see Molle and Kremer, 2010). Highlighting the degree of conservation in *Actinobacteria*, the genetic organization of the gene cluster encoding *pknA* and *pknB* is similar in *C. glutamicum*, *M. tuberculosis* and *S. coelicolor* (Boitel et al., 2003; Schultz et al., 2009). Along with cell division/cell wall biosynthesis genes, this cluster encodes two forkhead-associated (FHA) domain containing proteins. Studies in *M. tuberculosis* revealed that one of these FHA domain containing proteins (FhaA (Rv0020c)) negatively regulates the activity of MviN (a pseudokinase essential for peptidoglycan biosynthesis and growth), after PknB transphosphorylation (Gee et al., 2012). The second FHA domain containing protein (Rv0019c) is phosphorylated by PknA and has been proposed to interact with FtsZ, and hence has been renamed FtsZ interacting protein A, FipA (Sureka et al., 2010). The FHA domain of FipA is necessary for interaction with PknA and under oxidative stress conditions phosphorylated FipA is essential for FtsZ localization. The two FHA domain containing genes encoded on the *C. glutamicum pkn* cluster, (cg0064 (*fhaA*) and cg0063 (*fhaB*)) have not been addressed in much detail, however recently it was established that FhaB is phosphorylated by PknA and PknL, *in vitro* (Letek et al., 2012). It remains to be established if these *C. glutamicum* FHA domain containing proteins play a role in cell division and cell shape determination. Taken together, it appears that STPKs play a role in regulating cell growth and morphology, along with cell division in both *Corynebacteria* and *Mycobacteria*. Additionally, STPKs play a role in pathogenicity and cell survival (for review see Molle and Kremer, 2010).

## Conclusion and outlook

In recent years, advances have been made in understanding the intracellular organization and regulation of actinobacterial cells. Although all the components and regulatory systems involved in spatial and temporal regulation of cytokinesis have not been identified and characterized yet, it is clear that cell division differs in many ways to that of the long-standing model organisms. With the new era of live cell imaging and single cell analysis it will be possible to ascertain the role(s) of the putative *Corynebacterineae* cell division proteins.

**Figure 3:**
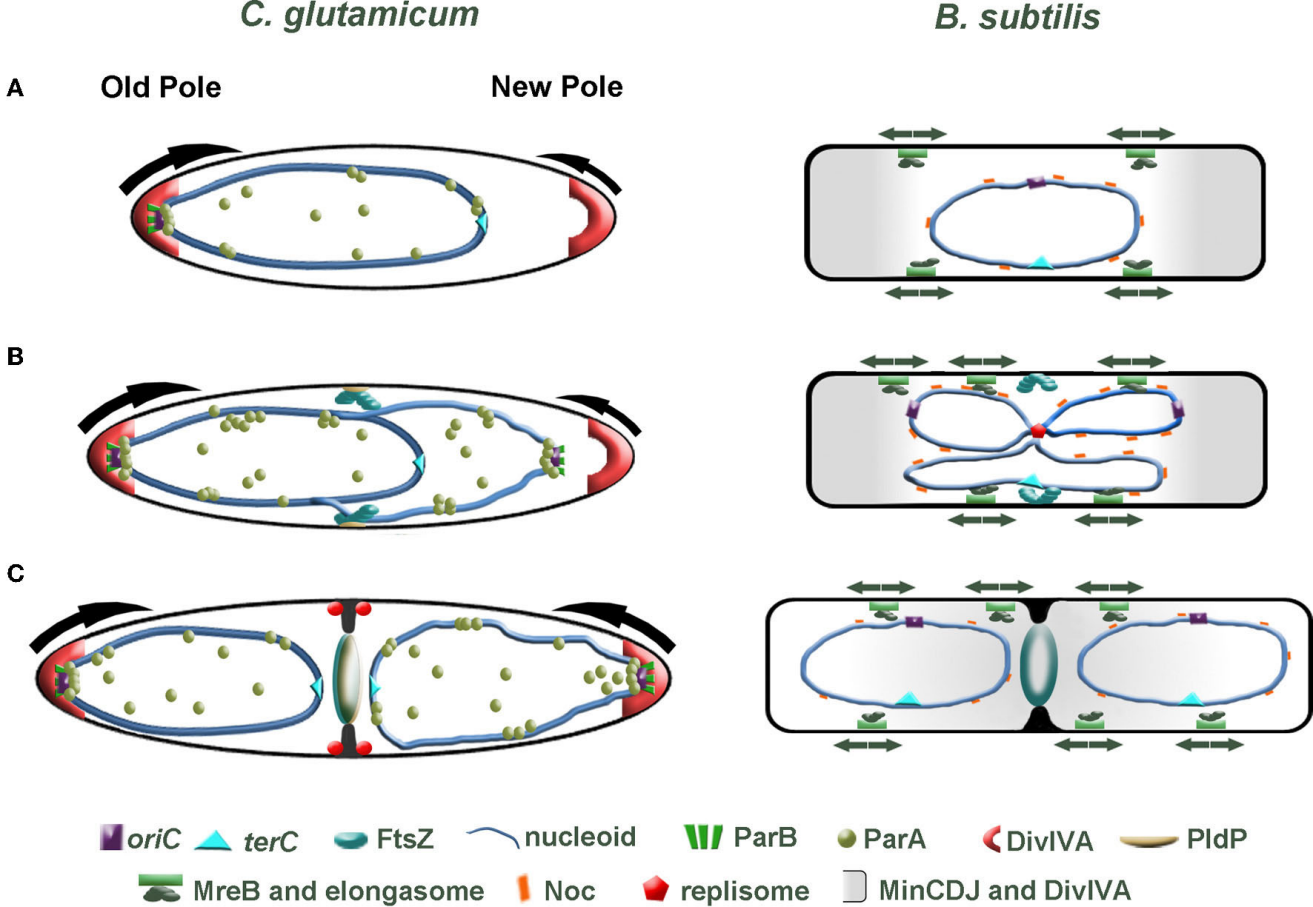
**Comparison of cell division in *C. glutamicum* and *B. subtilis*. (A)** Directly after cell division, one cell pole contains a ParB-bound origin that is tethered at the old cell pole through interaction with the polar growth determinant DivIVA. At this stage of the cell cycle, we speculate that polar growth is asymmetric with the pole lacking a tethered origin (young pole) growing slower than origin bound pole (arrows). In *B. subtilis*, the post-divisional cell contains a centrally located nucleoid with the *oriC* and *terC* also found positioned at the midcell. **(B)** Initiation of chromosome replication gives rise to duplication of the *oriC*. In *C. glutamicum*, the newly duplicated *oriC* is bound by ParB, then ParA is recruited and the *oriC* is segregated to the opposite cell pole. FtsZ assembles into a Z-ring prior to complete segregation of the chromosome. The Z-ring does not always assemble precisely at midcell. The orphan ParA-like protein, PldP localizes to the division site, where it might function to spatially regulate cell division. In *B. subtilis* a centrally positioned replisome duplicates the chromosome. While the Min system protects the poles and Noc protects the chromosome from aberrant Z-ring assembly, some aspect of replication initiation positively influences midcell localization of the Z-ring (note that DivIVA is not part of the Min system here). Contrary to the asymmetric polar growth in *C. glutamicum*, *B. subtilis* grow at a uniform rate along the lateral axis (arrows). **(C)** In *C. glutamicum*, the sister origin is tethered at the cell pole by an interaction with DivIVA. This interaction then leads to an increase in growth from this pole (arrow). Polar growth, in addition with bulk chromosome segregation mechanisms, would aid in segregating the chromosomes. As the septum invaginates, DivIVA begins to localize at the site of division. In *B. subtilis*, the Min system moves from the cell poles to the invaginating septum. The midcell localized Min system prevents the divisome from reassembling near to an old division site.

In new-born *C. glutamicum* cells, we speculate that the *oriC* is orientated toward the old cell pole, where it is tethered in position through an interaction between DivIVA and the ParB-*oriC* nucleoprotein complex. On the contrary, the *oriC* in *B. subtilis* and *E. coli* is found at midcell directly after division. There is an increasing body of evidence suggesting that replication initiation (and subcellular localization of the replication machinery) and midcell Z-ring assembly are coordinated. Using an outgrowing spore system to synchronize *B. subtilis* cells it was shown that Z-rings assemble asymmetrically at one side of the nucleoid when replication is completely inhibited or halted shortly after the *oriC* region has been replicated (Harry et al., 1999; Regamey et al., 2000). However, permitting replication initiation but blocking of DNA synthesis resulted in midcell assembly of Z-rings. From these observations it appears that the early stages of chromosome initiation positively influence midcell localization of FtsZ. In *C. glutamicum*, FtsZ assembles in the vicinity of the midcell region before the chromosomes have been completely segregated (Figure 3). It seems likely that the localization of the *oriC* and hence subcellular address of the replisome impact on the positioning of FtsZ. In line with this prospect, the positioning of division septa in *C. glutamicum* are more variable in cells lacking *parA* or *parB*, hinting that either the overall chromosome organization or segregation of the *oriC* regions might coordinate cell division and replication / segregation of the chromosomes. We additionally speculate that segregation of the *oriC* region to the opposite cell pole might influence the rate of polar growth at the new cell pole through. Recently, acyl-acyl carrier protein phosphate acyltransferase (PlsX) an enzyme involved in phospholipid and fatty acid synthesis in *B. subtilis* was identified as an interaction partner of FtsA and thus is a novel component of the divisome machinery (Takada et al., 2014). In the absence of *plsX*, Z-rings assemble at aberrant sites. More interestingly, the localization of PlsX is affected by DNA replication progression. Once again, the complexity of spatio-temporal regulation of cell division is highlighted. All in all, bacteria have evolved multiple, probably partially redundant mechanisms to regulate cell division. In *C. glutamicum*, the ParB-*oriC* nucleo-protein complex is positioned at the cell pole, where it is tethered by interaction with the polar growth determinant DivIVA. Given that reduced expression of *divIVA* phenocopies a *parB* deletion mutant, we speculate that anchoring the replicated origin at the cell pole influences the growth rate of the pole (Donovan et al., 2012) (Figure 3). Asymmetry in polar growth has been observed in *C. glutamicum* (Sieger et al., 2013). It will be interesting to determine if the slower growing pole is the pole void of an anchored origin.

### Conflict of interest statement

The authors declare that the research was conducted in the absence of any commercial or financial relationships that could be construed as a potential conflict of interest.
