# Corrigendum: Cell division in *Corynebacterineae*

**DOI:** 10.3389/fmicb.2016.01980

**Published:** 2016-12-15

**Authors:** Catriona Donovan, Marc Bramkamp

**Affiliations:** Department of Biology I, Ludwig-Maximilians-University of MunichPlanegg-Martinsried, Germany

**Keywords:** cell division, cell cycle, Par system, *Corynebacterium glutamicum*, *Mycobacterium tuberculosis*, FtsZ, DivIVA, serine/threonine kinases

We noticed that a wrong gene code was given for the *Corynebacterium glutamicum ftsZ* gene in Table 1 (page 3, first gene). The correct gene code for *C. glutamicum ftsZ* is cg2366.

The authors apologize for any inconvenience this mistake may have caused. This does not affect the scientific conclusions of the article.

## Conflict of interest statement

The authors declare that the research was conducted in the absence of any commercial or financial relationships that could be construed as a potential conflict of interest.

